# The *Drosophila* Cytosine-5 Methyltransferase Dnmt2 Is Associated with the Nuclear Matrix and Can Access DNA during Mitosis

**DOI:** 10.1371/journal.pone.0001414

**Published:** 2008-01-09

**Authors:** Matthias Schaefer, Julia P. Steringer, Frank Lyko

**Affiliations:** Division of Epigenetics, Deutsches Krebsforschungszentrum, Heidelberg, Germany; University of Birmingham, United Kingdom

## Abstract

Cytosine-5 methyltransferases of the Dnmt2 family are highly conserved in evolution and their biological function is being studied in several organisms. Although all structural DNA methyltransferase motifs are present in Dnmt2, these enzymes show a strong tRNA methyltransferase activity. In line with an enzymatic activity towards substrates other than DNA, Dnmt2 has been described to localize to the cytoplasm. Using molecular and biochemical approaches we show here that Dnmt2 is both a cytoplasmic and a nuclear protein. Sub-cellular fractionation shows that a significant amount of Dnmt2 is bound to the nuclear matrix. Sub-cellular localization analysis reveals that Dnmt2 proteins are enriched in actively dividing cells. Dnmt2 localization is highly dynamic during the cell cycle. Using live imaging we observed that Dnmt2-EGFP enters prophase nuclei and shows a spindle-like localization pattern during mitotic divisions. Additional experiments suggest that this localization is microtubule dependent and that Dnmt2 can access DNA during mitotic cell divisions. Our results represent the first comprehensive characterization of Dnmt2 proteins on the cellular level and have important implications for our understanding of the molecular activities of Dnmt2.

## Introduction

The methylation of cytosine residues plays an important role in the regulation of nucleic acids. Cytosine-5 RNA methylation is one among many different RNA modifications and has been detected in tRNA, rRNA and mRNA [1]. Cytosine-5 DNA methylation represents an important epigenetic modification regulating gene expression in eukaryotes and has been shown to be important during development and for the etiology of human disease [2], [3]. Elucidating the molecular mechanisms mediating RNA and DNA methylation is crucial to understand the roles that diverse nucleic acids play in the regulation of genetic information.

Based on the conservation of catalytic cytosine-5 DNA methyltransferase motifs, Dnmt2 has been assigned to the DNA methyltransferase enzyme family [4], [5]. Dnmt2 proteins have been widely conserved during evolution and their protein structure closely resembles that of known cytosine-5 DNA methyltransferases [6]. While initial studies failed to detect an enzymatic activity for Dnmt2, more recent reports have provided evidence for a low but significant DNA methyltransferase activity for human [7], *Drosophila* [8], *Entamoeba* [9] and Dictyostelium [10] Dnmt2. In addition, it has also been shown that Dnmt2 from various organisms methylates cytosine 38 in the anticodon loop of tRNA^Asp^ [11]. These data raised the possibility that the substrate specificity of Dnmt2 enzymes might be broader than previously anticipated [12].

Expression analyses of Dnmt2 in various model systems have suggested that Dnmt2 might be developmentally and tissue-specifically regulated. For example, human and mouse Dnmt2 have been shown to be expressed at relatively high levels in the heart, lung, kidney and testis [4], [5]. In addition, Dnmt2 expression has been shown to be elevated during early developmental stages in *Drosophila* and in zebrafish [8], [13], [14], which suggested a developmental role of the protein. In agreement with this notion, Dnmt2 mutant zebrafish showed defective liver, brain and retina development [15]. Understanding the significance of this phenotype, will require further investigation, because Dnmt2 mutant mice, flies and plants (*Arabidopsis thaliana*) have been described to be viable and fertile [11]. The limited understanding of the cellular characteristics of Dnmt2 has so far precluded a detailed analysis of Dnmt2 mutant organisms.

Because of their annotation as DNA methyltransferases, Dnmt2 enzymes have been expected to be nuclear proteins. However, ectopically expressed human Dnmt2 has been shown to localize in the cytoplasm of transiently transfected cells [11], which is in contrast to the nuclear localization of established DNA methyltransferases [16], and has been interpreted to be in agreement with the tRNA methyltransferase activity of the protein [11]. However, this observation cannot provide an explanation for the observed DNA methyltransferase activity of Dnmt2. In addition, a Dnmt2 homologue in Dictyostelium has been shown to reside in the nucleus [10] and a Dnmt2 homologue in *Entamoeba* has been associated with the nuclear matrix [17]. These observations are difficult to reconcile with the conclusion that Dnmt2 is exclusively cytoplasmic [11].

Because the identification of sub-cellular compartments associated with individual proteins is important for understanding their molecular activities, a systematic analysis of the sub-cellular localization of Dnmt2 should provide valuable information to define the function of these enzymes. In order to characterize Dnmt2 in *Drosophila* in more detail we created specific antibodies to biochemically trace Dnmt2 as well as fusion proteins to EGFP and GAL4:VP16, that allowed us to study the sub-cellular dynamics and localization of Dnmt2. We show that Dnmt2 is also a nuclear protein, which is part of the insoluble nuclear matrix. Dnmt2-EGFP could be predominantly visualized in endo-replicating and dividing nuclei. These findings show that the sub-cellular distribution of Dnmt2 is fundamentally different from that of other DNA or tRNA methyltransferases and provide experimental support for the notion that Dnmt2 enzymes have multiple molecular activities.

## Results

### Expression of Dnmt2 during Drosophila development

**Figure 1:**
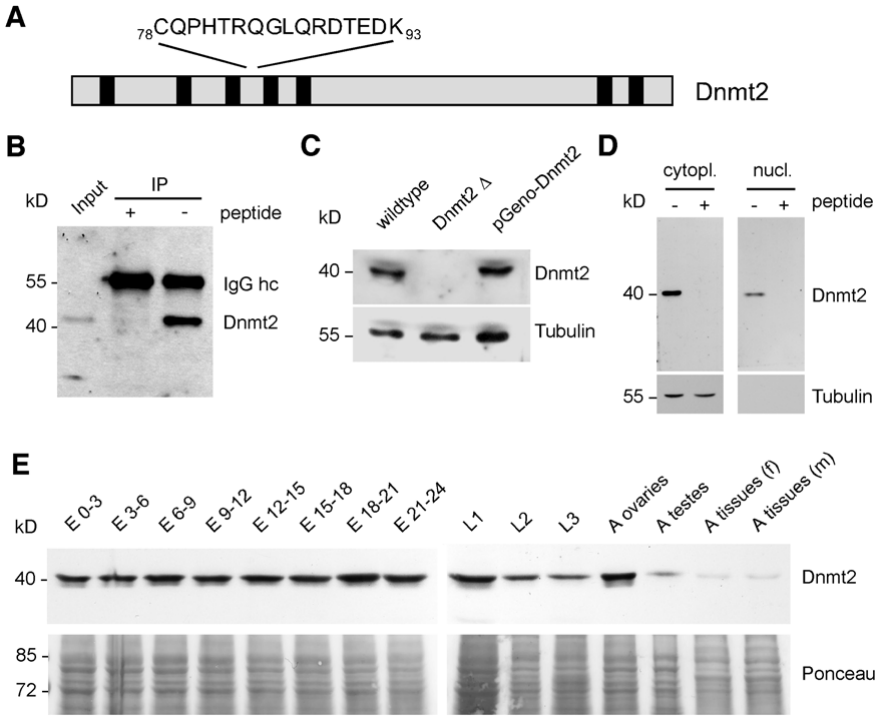
Establishment of Dnmt2-specific antibodies and characterization of Dnmt2 expression during *Drosophila* development. (A) Sequence of the epitope recognized by polyclonal antibodies against Dnmt2 peptide spanning amino acids 78–93. (B) Affinity-purified anti-Dnmt2 antibodies efficiently immunoprecipitate Dnmt2 from whole cell protein extracts. (C) Anti-Dnmt2 specifically detects a 40 kDa protein. Whole-cell protein extracts from wildtype, homozygous mutants (Dnmt2Δ) and rescued mutants (pGenoDnmt2) were blotted and probed with anti-Dnmt2. (D) Early embryonic tissue (0–6 hr AEL) was fractionated, blotted as cytoplasmic and nuclear fractions and probed with anti-Dnmt2 and anti-〈-tubulin as control. (E) Developmental Western blot. Wildtype cytoplasmic protein extract from various embryonic, larval and adult stages were blotted and probed with anti-Dnmt2. Ponceau staining of blot is shown as loading control. hc-heavy chain, cytopl.-cytoplasmic, nucl.-nuclear, E-embryo, L1-first instar larvae, L2-second instar larvae, L3-third instar larvae, A-adult, f-female, m-males.

As an initial step towards the characterization of Dnmt2, we affinity purified antibodies against a peptide epitope encompassing amino acids 78–93 of the annotated protein (Mt2-PA, Genebank accession no. AAF53163) (Fig. 1A). These antibodies recognize Dnmt2 as a 40 kDa protein on Western blots and can efficiently immunoprecipitate Dnmt2 from protein extracts, as confirmed by mass-spectrometry (Fig. 1B and data not shown). The 40 kDa polypeptide was not recognized in protein extracts from a homozygous Dnmt2 mutant strain [11]. The introduction of a genomic Dnmt2 transgene (pGeno-Dnmt2) into the Dnmt2 mutant background via P-element mediated germ line transformation led to the re-expression of the 40 kDa polypeptide (Fig. 1C). The available experimental data indicates that Dnmt2 can methylate both DNA and tRNA. This suggests that the protein should be detectable not only in the cytoplasm but also in nuclei. We therefore biochemically fractionated 0–6 hours old wildtype embryos to obtain cytoplasmic and nuclear protein extracts and found that the majority of Dnmt2 protein is cytoplasmic. However, purified nuclei contained a significant amount of Dnmt2 (Fig. 1D). Furthermore, testing protein extracts of wildtype animals of various developmental stages we observed that Dnmt2 is expressed throughout development and in adults. (Fig. 1E). We concluded that Dnmt2 is present during all stages of *Drosophila* development and that a fraction of cellular Dnmt2 resides in nuclei.

### Dnmt2 is expressed in specific tissues during embryogenesis and in the adult germ line

**Figure 2:**
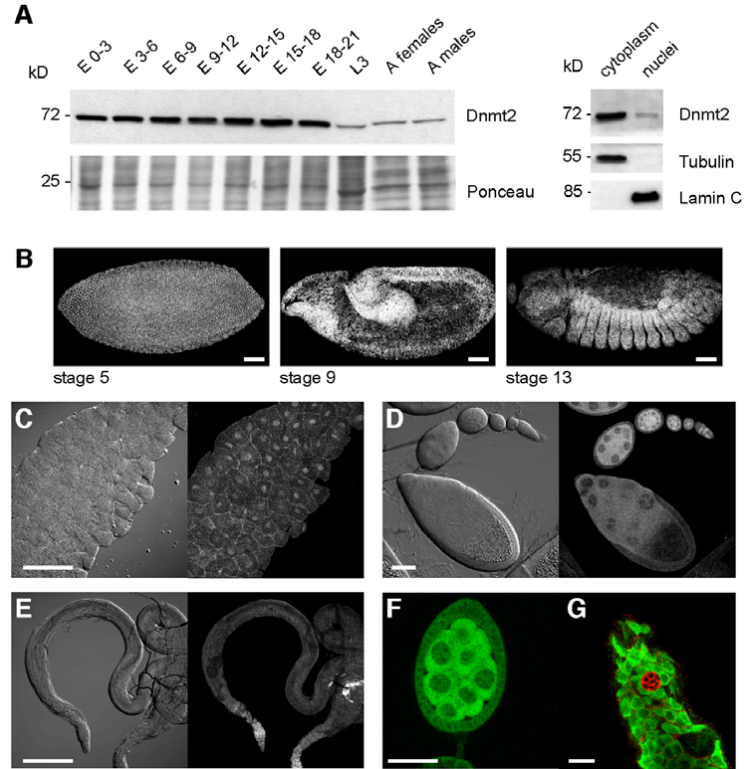
Tissue specific expression of Dnmt2. (A) Left panel: developmental Western blot of pGeno-Dnmt2-EGFP animals. Whole protein extract from embryonic, larval and adult stages were blotted and probed with anti-Dnmt2. Ponceau staining is shown as a loading control. Right panel: Dnmt2-EGFP is expressed both in the cytoplasm and in nuclei. Fractionation efficiency was confirmed by probing the blot for cytoplasmic 〈-tubulin and nuclear Lamin C. Antibodies against EGFP reveal Dnmt2 in developing embryos (B), third instar larval salivary glands (C), ovaries (D) and testes (E). (F) Dnmt2-EGFP in a developing cyst of the female germline is mostly cytoplasmic. Nuclear staining can be observed in nurse cell nuclei. (G) Dnmt2-EGFP in the male germline is ubiquitous in all cells with the exception of somatic hub cells (marked by Armadillo, red). Scale bars: (B) 100 µm; (C) 10 µm; (D) 50 µm; (E) 100 µm; (F) 20 µm; (G) 10 µm.

Since Dnmt2 expression could be detected during all stages of *Drosophila* development we asked whether Dnmt2 displays a tissue-specific expression. Our affinity purified antibodies against Dnmt2 were not suitable for indirect immunofluoresence experiments, which was most likely due to epitope masking (data not shown). We therefore created several independent transgenic fly lines harbouring a Dnmt2-EGFP fusion gene in the genomic sequence context of the Dnmt2 locus (pGeno-Dnmt2-EGFP). Since Dnmt2 mutant animals are viable and fertile and do not display obvious phenotypes we could not positively test whether the genomic Dnmt2-EGFP contstruct is functional. On the other hand, co-immunoprecipitation experiments using both endogenous Dnmt2 as well as Dnmt2-EGFP constructs revealed that Dnmt2-EGFP is contained in similar protein complexes, suggesting that part of the functionality of Dnmt2 is retained in the fusion protein (unpublished observations). In order to test whether these constructs express tagged Dnmt2 similarly to endogenous Dnmt2 we performed Western blot analysis of protein extracts from various developmental stages and adult tissues using antibodies against Dnmt2. We found that Dnmt2-EGFP was expressed during all stages of development and at levels that were comparable with those of the endogenous Dnmt2 protein (Fig. 2A). Furthermore, biochemical fractionation of embryonic protein extracts and immunolocalization of Dnmt2-EGFP in salivary glands showed that the fusion protein could be imported into nuclei, suggesting that the sub-cellular localization signals were functionally retained (Fig. 2A and C).

To further validate the expression of the pGeno-Dnmt2-EGFP construct we stained embryos and adult tissue using anti-EGFP antibodies. We found that the construct was ubiquitously expressed during early embryogenesis. Expression remained ubiquitous throughout development with enrichment in the fore- and hindgut as well as in the developing mesoderm in gastrulating embryos (Fig. 2B). This expression pattern strongly coincided with the published pattern of the endogenous Dnmt2 transcript [14], which further indicated that the pGeno-Dnmt2-EGFP construct mirrors the endogenous expression pattern of Dnmt2 transcripts.

Since endogenous Dnmt2 is expressed in ovaries and testes (Fig. 1D) we determined the cellular Dnmt2-EGFP protein expression pattern during gametogenesis using anti-EGFP antibodies. We found that Dnmt2-EGFP is highly expressed in the germarium, blastocyst and cyst stages of the female germ line with the noticeable exception of follicle cells (Fig. 2D). Dnmt2-EGFP was mostly present in the cytoplasm but could also be found in nurse cell nuclei (Fig. 2D, F). In the male germ line, Dnmt2-EGFP was expressed in most cells, including stem cells as well as in maturing spermatocytes (Fig. 2E) with the exception of the post-mitotic hub cells (Fig. 2G). Dnmt2-EGFP protein was mostly cytoplasmic and mature sperm did not show any Dnmt2-EGFP signal above background (data not shown). From these observations we concluded that Dnmt2-EGFP is expressed in DNA-synthesizing tissues such as endo-replicating nurse and salivary gland cells as well as in the proliferating regions of the adult germ line.

### A significant fraction of Dnmt2 localizes to nuclei

**Figure 3:**
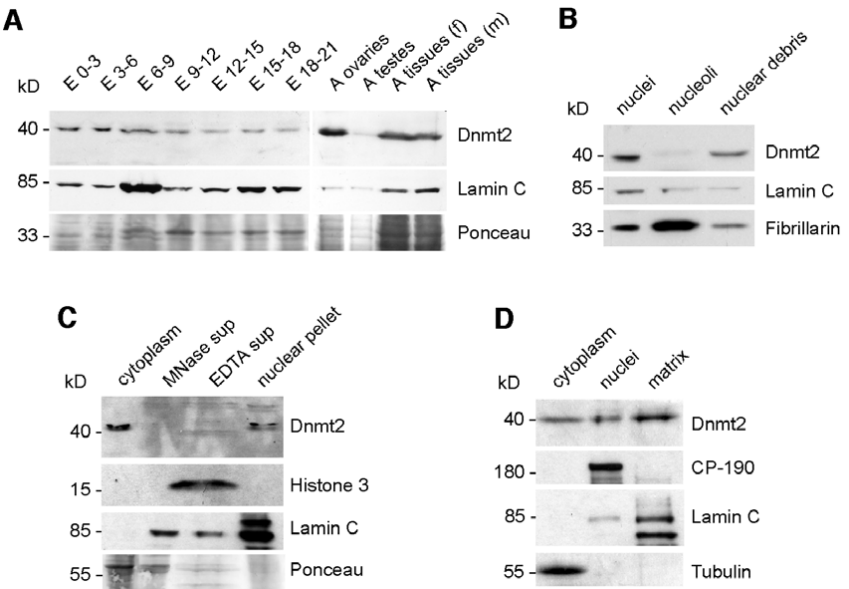
Dnmt2 is present in nuclei and resides in an insoluble part of the nuclear matrix. (A) Developmental Western blot. Purified nuclei from embryonic and adult tissues were urea extracted, blotted and probed with anti-Dnmt2. Ponceau staining is shown as a loading control, lamin C staining is shown as a control for the enrichment of nuclear proteins. (B) Dnmt2 is not enriched in nucleoli. Sucrose density fractionation of nucleoli followed by blotting and tracing of nucleolar material using anti-Fibrillarin shows that Dnmt2 is part of other nuclear structures. (C) Dnmt2 is not enriched in nucleosomes. Micrococcal nuclease digest of purified nuclei releases histone H3 (EDTA supernatant), but not Dnmt2. The EDTA extracted nuclei still contain Dnmt2 suggesting association of Dnmt2 with non-chromatin structures. (D) Dnmt2 is associated with the nuclear matrix. Nuclear Dnmt2 is insoluble after DNAse digest, followed by high salt extraction and can only be solublilzed using urea extraction. CP-190 is included as a peripheral and lamin C as an integral nuclear matrix interacting protein. E-embryo, A-adult, f-female, m-males, MNase-micrococcal nuclease, sup-supernatant.

Since a fraction of Dnmt2 could be purified with intact nuclei we asked whether this nuclear localization could be developmentally regulated. Western blots of purified nuclei show that a significant amount of Dnmt2 protein can be recovered in all developmental stages analyzed (Fig. 3A). Nuclear Dnmt2 protein levels appeared to be comparably elevated in young (E 0-3, E 3-6) embryos and in ovaries (Fig. 3A).

Because tRNA modifications have been reported to occur in nucleoli [18], [19], we sought to identify the sub-nuclear structures associated with Dnmt2. As a first step, we tested whether Dnmt2 co-fractionates with nucleoli. After disruption of purified nuclei and density fractionation through sucrose cushions we separated nucleoli from the rest of the nuclear structures. Tracing nucleolar material using an antibody against fibrillarin we found that Dnmt2 was not enriched in nucleoli (Fig. 3B). Using micrococcal nuclease (MNase) digestion of purified nuclei to release histone-associated chromatin, we observed that nuclear Dnmt2 is not solubilized, suggesting that Dnmt2 is not chromatin-bound. While canonical histones such as histone H3 were released from MNase and EDTA treated nuclei, Dnmt2 remained in the nuclear remnants (Fig. 3C). To determine whether the nuclear Dnmt2 fraction is associated with the nuclear matrix we salt extracted purified nuclei. For controls we used antibodies against the insulator protein CP-190, which is peripherally associated with the nuclear matrix, as well as antibodies against Lamin C that is an integral part of the nuclear matrix. These experiments showed that most of the nuclear Dnmt2 protein remains insoluble after extraction. In order to analyze nuclear Dnmt2, salt-extracted nuclei had to be treated with 7 M urea to solubilize the protein (Fig. 3D). We concluded that nuclear Dnmt2 is not available as a soluble protein in the nucleoplasm but is part of insoluble structures, such as the nuclear matrix.

### Dnmt2 is enriched in mitotic cells

**Figure 4:**
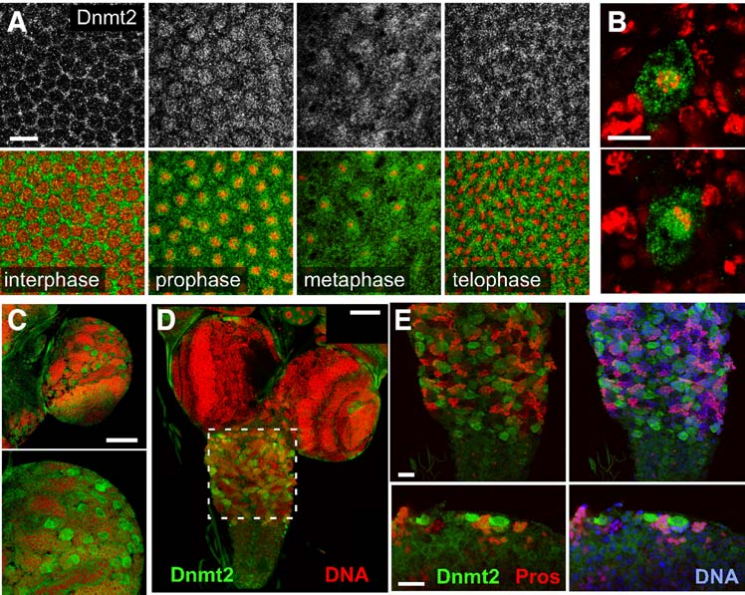
Dnmt2 localizes to mitotically active cells. (A) pGeno-Dnmt2-EGFP localizes to mitotic nuclei during prophase and localizes to midbody structures in telophase as visualized by anti-EGFP staining. Pictures show the distribution of Dnmt2-EGFP (gray, upper panel) and merged images (lower panel) with Dnmt2-EGFP in green and DNA in red. (B) Ectopic Dnmt2-EGFP localizes around metaphase chromosomes. Cell-type specific expression of UAS-Dnmt2-EGFP using asense-GAL4 causes Dnmt2 to localize to nuclear structures during mitosis. Ectopic protein localizes to prophase chromatin of CNS neuroblasts (upper panel) and aggregates densely around the mitotic metaphase plate (lower panel). (C) pGeno-Dnmt2-EGFP localizes to mitotic nuclei during larval development (upper panel). Frequently dividing optic lobe neuroblasts are enriched for Dnmt2-EGFP expression as visualized by anti-EGFP staining (lower panel). (D–E) Ventral ganglion neuroblasts express high levels of Dnmt2-EGFP, whereas ganglion mother cells (marked by Prospero, red, in E), which give rise to differentiated neurons and glia, do not express Dnmt2-EGFP. Scale bars: (A) 10 µm; (B) 10 µm; (C, D) 50 µm; (E, F) 20 µm; (G) 10 µm.

Dnmt2 can reside both in nuclei and in the cytoplasm, which suggests that the protein is shuttling between these compartments. We therefore sought to characterize the dynamic aspects of Dnmt2 localization. However, the comparably low expression levels of the genomic Dnmt2-EGFP reporter construct precluded the direct visualization of Dnmt2-EGFP in embryos. Additionally, yolk autofluorescence did not allow for high resolution imaging in embryonic tissues. We therefore determined the localization of Dnmt2-EGFP using anti-EGFP antibodies in fixed cleavage stage embryos and found that Dnmt2-EGFP accumulated around nuclear structures at the beginning of M-phase during the nuclear division cycles. This protein localization persisted into anaphase, when Dnmt2-EGFP became concentrated at structures between daughter nuclei and returned to become cytoplasmic at the time of karyokinesis (Fig. 4A).

We next determined whether Dnmt2 localizes to mitotic nuclei in developmental stages beyond the syncitial cleavage divisions. Ectopic expression of Dnmt2 during later developmental stages using both ubiquitous (hs-GAL4, data not shown) and cell-specific promoters driving GAL4 (asense-GAL4) in flies carrying a UAS-Dnmt2-EGFP transgene we observed that the Dnmt2 fusion protein also accumulated around chromosomes in mitotic cells, such as in neuroblasts in the embryonic neuroectoderm (Fig. 4B). In contrast, actin5C driven UAS-EGFP protein showed a diffuse cytoplasmic localization under the same experimental conditions (Fig. S1) suggesting that the Dnmt2 mojety targets the EGFP signal to mitotic chromatin.

In order to further analyze the mitotic localization of Dnmt2 we used the developing larval brain, which contains a high number of proliferating neuroblasts giving rise to ganglion mother cells (GMC). Analyzing third instar larval brains of animals expressing pGeno-Dnmt2-EGFP (Fig. 4C, D) we found EGFP positive cells marking dividing neuroblasts whereas Dnmt2-EGFP was absent from GMC that were identified by staining for the transcription factor Prospero (Fig. 4E). From these results we concluded that Dnmt2 is enriched in mitotic but not in post-mitotic cells, which suggests a role of Dnmt2 in actively cycling cells.

### Dnmt2 is dynamically localizing to mitotic nuclei

To study the localization of Dnmt2 in living tissue we used transgenic flies expressing a Dnmt2-EGFP fusion protein under the control of the ubiquitin promoter (pUbq-Dnmt2-EGFP). This allowed for sufficiently high levels of Dnmt2-EGFP in early embryos and overcame the technical problems described previously. To follow nuclear divisions we crossed Dnmt2-EGFP expressing flies with animals expressing the histone H2A variant fused to red fluorescent protein (His2Av-mRFP1) which served as a chromatin marker.

**Figure 5:**
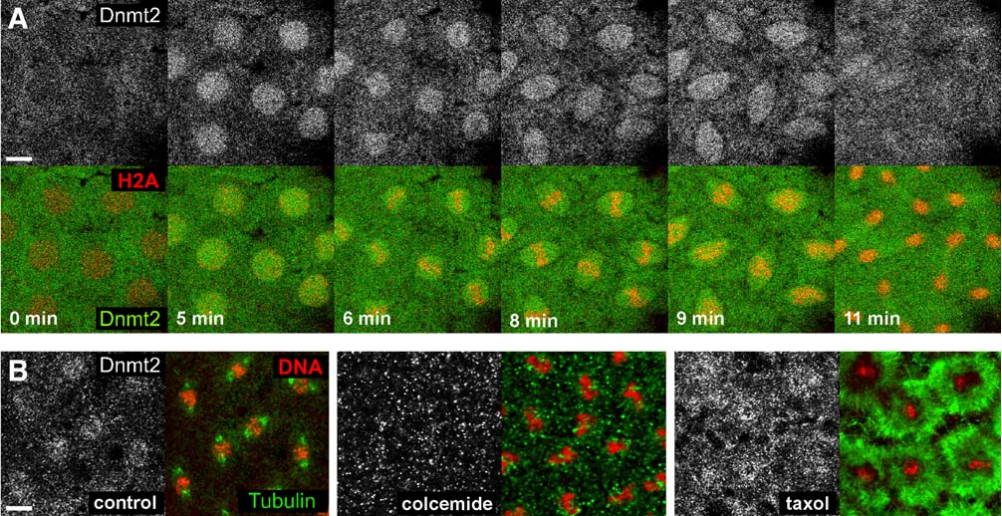
Dnmt2 localization to mitotic nuclei is dynamic and microtubuli-dependent. (A) Nuclear division cycle 10 embryos expressing pUbq-Dnmt2-EGFP and His2Av-mRFP1 were mounted on an open coverslip and viewed by dual wavelength time-lapse LSCM. Selected frames are shown for Dnmt2-EGFP (gray, upper panel) and as merged images (lower panel) with Dnmt2-EGFP in green and His2A-mRFP1 in red. A movie of the sequence is available as supporting material. (B) 1–3 h old embryos expressing pGeno-Dnmt2-EGFP were collected and fixed immediately (left panel), treated with colcemide (middle panel) or with taxol (right panel) in order to disrupt or stabilize microtubuli, respectively. Metaphase chromosomes are shown with Dnmt2 (gray), DNA (red) and 〈-tubulin (green). While Dnmt2-EGFP is maintained around metaphase plates in taxol-treated embryos, the protein is delocalized in colcemide-treated embryos. Scale bars: (A) 10 µm; (B) 5 µm.

Imaging Dnmt2-EGFP through consecutive cleavage cycles revealed that the fusion protein is distributed diffusely during S-phase but rapidly localizes to prophase nuclei at the onset of mitosis. During meta- and anaphase Dnmt2-EGFP formed spindle-like structures and re-localized to midbody structures during karyokinesis. During the following S-phase Dnmt2-EGFP was diffusely distributed throughout the syncitial cytoplasm until the start of the next mitotic phase. (Fig. 5A and movie S1).

The mitotic localization of Dnmt2 was reminiscent of microtubule (MT) structures that form the spindle apparatus during cell divisions. We co-stained embryos for Dnmt2-EGFP and tubulin and found that the signals overlap only partially (Fig. 5B-left panel). In order to test whether Dnmt2-EGFP localization depends on the presence of intact MTs we treated embryos with colcemide to destabilize MTs. We found that the spindle-like Dnmt2-EGFP localization in mitotic nuclei was lost (Fig. 5B-middle panel). Stabilization of MTs using taxol caused a more diffuse localization of Dnmt2-EGFP around mitotic chromosomes (Fig. 5B-right panel) but did not trigger the re-localization of Dnmt2-EGFP to ectopic microtubule-rich regions, indicating an indirect interaction between Dnmt2-EGFP and MTs. We concluded that the syncitial pool of Dnmt2-EGFP is dynamically changing from a uniformly cytoplasmic localization during S-phase to a microtubule-dependent spindle-like distribution during pro- and metaphase of mitosis.

**Figure 6:**
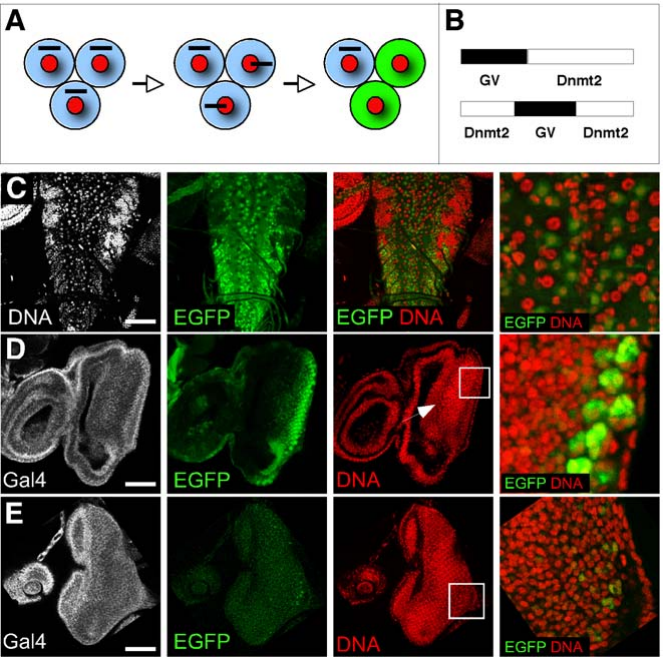
Nuclear access reporter assay using GAL4:VP16-Dnmt2 fusion proteins. (A) Schematic representation of the nuclear access reporter assay. Ubiquitous expression of the GAL4:VP16 (GV) fusion proteins (black bar) causes the expression of EGFP only in some cells (green) due to selective nuclear access of the fusion protein. (B) GV-fusion proteins. GV-Dnmt2 (upper panel), Dnmt2-GV-Dnmt2 fusion expressing GV in the context of a split Dnmt2 protein (lower panel) which served as control. (C) GAL4:VP16-Dnmt2 expression in third instar larval brains causes widespread UAS-EGFP expression in the ventral ganglion. The magnification shows that regularly spaced cells (neuroblasts) express EGFP (green). (D) Ubiquitous GAL4:VP16-Dnmt2 in third instar larval eye discs causes specific UAS-EGFP expression behind the morphogentic furrow (arrow). Magnification of a field of cells shows robust expression in differentiated photoreceptor cells. (E) The control construct (GAL4:VP16 in a split Dnmt2 protein) causes background levels of UAS-EGFP expression. GAL4 (gray), EGFP (green), DNA (red). Scale bars: (C) 100 µm; (D, E) 70 µm.

The dynamic localization of Dnmt2 to mitotic chromosomes raised the possibility that Dnmt2 could transiently access DNA during this particular phase of the cell cycle. In order to test whether Dnmt2 is interacting with DNA we used a chimeric transcription factor GAL4:VP16 (GV) fused to Dnmt2. The GAL4-VP16 protein contains the DNA-binding domain of the yeast transcription factor GAL4 linked to the transcriptional activating domain of the viral VP16 protein [20]. This reporter system has been used previously to characterize the nuclear access of the Notch intracellular domain [21] and was therefore be adapted to trace the nuclear access of GV-Dnmt2 (Fig. 6A, B). Because overexpression of GAL4-VP16 alone is cytotoxic to cells (G. Struhl, personal communication) we also established a control construct where the GAL4-VP16 domain was embedded in a split Dnmt2 protein (Fig. 6B). The resulting GV-Dnmt2 proteins were ubiquitously expressed in animals also carrying a UAS-EGFP transgene. Expression of GV-Dnmt2 from the polyubiquitin promotor in embryos showed ubiquitously high levels of UAS-EGFP (data not shown). We therefore focused our analysis on larval imaginal disc tissues that develop more slowly and contain cycling and differentiating cells. In the larval brain we observed transactivation of the EGFP reporter in many cells interspersed by cells not displaying EGFP signal (Fig. 6C). This pattern was reminiscent of the expression pattern of Dnmt2-EGFP expressed from the genomic construct (Fig. 4D,E) suggesting that cycling neuroblasts allow access of the transactivator fusion protein to DNA. In order to directly examine the possibility that mitotic cells allow access of Dnmt2 to DNA we analyzed third instar larval eye discs. These tissues undergo a well-characterized differentiation process after a mitotic wave of cell divisions (morphogenetic furrow). Even though both Dnmt2 fusion proteins were expressed ubiquitously across the eye discs (Fig. 6D,E-GAL4 panel), we observed that GAL4-VP16 access to DNA occured only with the GV-Dnmt2 construct, as evidenced by a strong EGFP expression in the differentiating photoreceptor cells (Fig. 6D). The control construct showed only weak and patchy activation of the UAS-EGFP reporter behind the morphogenetic furrow reflecting the stochastic background access of GAL4-VP16 to DNA (Fig. 6E). These experiments suggest that Dnmt2 can access DNA in cells undergoing mitosis and thus defines a cell cycle stage that would allow Dnmt2 to interact with genomic DNA under *in vivo* conditions.

## Discussion

Dnmt2 proteins are highly conserved enzymes that function as cytosine-5 methyltransferases. Their ability to methylate both DNA and RNA distinguishes Dnmt2 enzymes from other cytosine-5 methyltransferases, and might provide a mechanistic link between RNA and DNA modifications. Proteins belonging to the Dnmt family of enzymes have traditionally been viewed to reside in the nucleus. Interestingly, cytoplasmic localization of Dnmt1 has also been described [22], but the functional significance of this observation is not understood. While DNA is concentrated in nuclei, RNA molecules are abundant both in nuclear and cytoplasmic compartments. Therefore, a cytosine-5 methyltransferase with activity towards both DNA and RNA substrates could function in any compartment containing nucleic acids and the specificity to the respective substrate could be determined by binding partners. Consistent with this notion, our results show that the localization of *Drosophila* Dnmt2 is both cytoplasmic and nuclear and therefore more complex than previously anticipated [11].

In contrast to previous work, which used either unpurified antibodies against Dnmt2 peptides [8] or antibodies cross-reacting with the catalytic motifs of mammalian Dnmt1 [13], we have used purified antibodies against a Dnmt2-specific peptide to unambiguously identify Dnmt2 as a 40 kDa cytoplasmic and nuclear protein. Both cellular compartments contain Dnmt2 throughout development. At the tissue level, Dnmt2 is ubiquitously expressed but is enriched in muscle and gut of the developing embryo, in the cytoplasm of developing ovarian cysts as well as in proliferating cells in testes. A tissue-specific expression pattern has also been observed for zebrafish Dnmt2 [15]. In contrast to zebrafish, the *Drosophila* null mutants for Dnmt2 are viable and fertile [11]. The reasons for this discrepancy are presently unknown.

It has been shown that epitope-tagged human DNMT2 localizes to the cytoplasm of transiently transfected NIH3T3 cells and this observation has been interpreted to reflect a general cytoplasmic localization of Dnmt2 [11]. However, a nuclear or chromatin localization of Dnmt2-like proteins has been reported in Dictyostelium [10] and in *Entamoeba*. In the latter organism, Dnmt2 has been biochemically associated with the nuclear matrix [17]. These results suggested that Dnmt2 proteins could interact directly with DNA. Of note, Dnmt2 contains no nuclear localization signal but a predicted nuclear export signal (aa 281-293). This suggests that other pathways are mediating Dnmt2 localization to nuclei and that nuclear occupancy might be time controlled. In agreement with this notion we found that Dnmt2 is re-localizing to nuclear structures during specific stages of the cell cycle.

Our sub-cellular fractionation experiments showed that a significant amount of nuclear Dnmt2 was insoluble after micrococcal nuclease digest, DNAse treatment and salt extraction. This suggested that nuclear Dnmt2 is tightly bound to structures such as the nuclear matrix. The nuclear matrix as a non-chromosomal scaffold within the nucleus remains a controversial concept in cell biology [23] and is poorly defined in terms of composition and function. Biochemically purified nuclear matrix material contains proteins, DNA and RNA [24]. Matrix attachment regions (MARs) in DNA are thought to separate chromatin into topologically constrained loop domains allowing to control for the compaction of chromatin as well as for DNA replication and gene regulation [25], [26]. The *Entamoeba* Dnmt2 homologue binds *in vitro* to EhMRS2, a DNA element containing the consensus scaffold/matrix attachment region (S/MAR) bipartite recognition sequences suggesting that Dnmt2 like molecules can interact directly with DNA [17]. EhMRS2 contains internal tandem repeats coding for small RNAs of unknown function [27]. The association of Dnmt2 with the nuclear matrix suggests that this cytosine-5 methyltransferase contributes to the regulatory functions of DNA elements that contain S/MAR sequences.

Live imaging analysis revealed that Dnmt2-EGFP is ubiquitously localized in the syncitial cytoplasm but condenses around mitotic chromosomes. This is reminiscent of the mitotic scaffold proteins Skeletor [28] and Chromator [29]. Similar to Dnmt2, these proteins form an intranuclear spindle-shaped structure during prophase that precedes the assembly of the spindle. Skeletor/Chromator and microtubules display a similar distribution throughout mitosis, but by telophase, Skeletor/Chromator re-associate with chromatin while Dnmt2-EGFP localizes to microtubule structures such as the midbody. Components of the spindle matrix have been hypothesized to be involved in the regulation of the nuclear architecture [30] and may provide structural support for the mitotic spindle [28].

The ability of Dnmt2 to methylate both DNA and tRNA predicts a complex localization pattern for this class of enzymes. While DNA methylation would be catalyzed in the nucleus, tRNA modifications have been shown to occur both in nucleoli and on the surface of the nuclear envelope in yeast [18]. Also, cytosine-5 methylation of rRNA has been shown to occur in nucleoli [31]. In agreement with these observations, the vertebrate Misu/NSUN2 protein, a cytosine-5 methyltransferase with catalytic activity towards DNA, tRNA and rRNA has been shown to localize to the nucleoplasm and to nucleoli [32], [33]. Interestingly, Misu/NSUN2 also localized to mitotic spindles during mitosis [32] and has been shown to be a substrate of aurora B kinase [33] which is essential for the segregation of chromosomes and an integral part of the mitotic passenger complex. It is possible that the mitotic localization pattern of Dnmt2 reflects the distribution of the protein into daughter cells via nuclear envelope vesicles [34]. However, the ability of Dnmt2 fusion proteins to access a genomic reporter construct in mitotic cells argues that the protein might be able to modify genomic DNA or nuclear RNAs during this stage.

## Materials and Methods

### Fly stocks

The Dnmt2 mutant allele has been described previously [11]. *yw* was used as a wild-type strain. Hs-GAL4 [35] or asense GAL4 (provided by Helen Skaer) were used for expression of UAS-Dnmt2-EGFP transgenes. UAS-EGFP transgenic flies were provided by Renato Paro, His2A-mRFP1 transgenic flies have been described previously [36]. Flies were reared on standard *Drosophila* medium at 25°C.

### DNA constructs

The construct for GAL4-inducible Dnmt2-EGFP expression was generated by replacing H2B in pH2B-EGFP (a gift from Peter Becker) with the ORF of Dnmt2. The construct was subsequently sub-cloned into pUAST [35] or pWR-Ubq [37]. pGeno-Dnmt2-EGFP was created by replacing the C-terminal half of Dnmt2 in a genomic Dnmt2 construct with a fragment containing the C-terminal half of Dnmt2 fused to EGFP from pUAST-Dnmt2-EGFP. pGeno-Dnmt2-EGFP encompasses 300 bp upstream of the start and 150 bp downstream of the stop codon of Dnmt2. This was sub-cloned into pCaSpeR-4. The construct containing the GAL4:VP16 fusion to Dnmt2 was created by subcloning the GAL4:VP16 domain from pNT63.10 [21] into a pbGlo2xmyc vector [38] containing the Dnmt2 cDNA to create an N-terminal in frame fusion protein of GAL4:VP16 to Dnmt2, followed by sub-cloning into pWR-Ubq. Constructs were verified by sequencing and expression analysis. Detailed information will be provided upon request. Transgenic fly lines were generated by P-element mediated germ line transformation (Rubin and Spradling, 1982).

### Antibodies

Polyclonal antibodies against Dnmt2 peptide epitopes were raised in rabbits and have been described elsewhere [8]. A Dnmt2 peptide (_78_CQPHTRQGLQRDTEDK_93_) was synthesized and coupled to SulfoLink gel (Pierce), according to the manufacturer's instructions. Peptide-specific antibodies were purified using high-salt conditions and elution in 0.1 M glycine (pH 3.5) followed by dialysis. Affinity purified Dnmt2 antibody was diluted 1∶500 for Western blots. Other antibodies used in Western blots (WB) or immunofluorescence (IF) were: armadillo (Hybridoma bank-N2 7A1) 1∶100 for IF; α-tubulin (Sigma DM1A), 1∶10.000 for WB and 1∶200 for IF, lamin C [39], 1∶1.000 for WB; EGFP (Abcam-Ab6556), 1∶2.000 for WB and 1∶1000 in IF; Prospero (Hybridoma bank-MR1A) 1∶100 for IF; fibrillarin (Abcam-Ab5821), 1∶1000 for WB, Histone 3 (Cell Signaling) 1∶10.000 for WB, CP190 [40] 1∶500 for WB, GAL4 (Upstate) 1∶500 in IF.

### Immunofluorescence

Embryo immunostainings were performed essentially as described [41], with the following modifications. For colcemide and taxol experiments, 1–3 h old embryos were treated with colcemide (5 mM) or taxol (25 µM) for 10 minutes before fixation as described previously [42]. After de-vitellinization in methanol, embryos were washed in PBS/0.1% Tx-100 and blocked in PBS/3% normal goat serum/3% BSA/0.1% Tx-100. Larval imaginal discs and brains were dissected from third instar larvae in PBS, fixed in 5% PFA, followed by treatment as used in embryo stainings. For ovary and testis stainings, well-fed adult flies were dissected in PBS and tissues were transferred into 5% PFA in buffer B (100 mM KH_2_PO4/K_2_HPO_4_ (pH 6.8), 450 mM KCl, 150 mM NaCl, 20 mM MgCl_2_) and fixed for 20 minutes at room temperature (RT), followed by treatment as used in embryo stainings. Fixed tissue was incubated overnight at 4°C with primary antibodies followed by incubation with secondary antibodies. Cy5 anti–mouse (Jackson ImmunoResearch Laboratories) were used 1∶250 and Alexa 488 anti–rabbit (Molecular Probes) antibodies were used at 1∶500. RNase A was included in procedure to 2 mg/ml for 1 hour if propidium iodide was used to stain DNA (Gonzalez and Glover, 1993). DNA was stained with DAPI (1 ng/ml) or propidium iodide (0,5 µg/ml, Molecular Probes). After mounting in Fluoromount (Biozol), samples were analyzed by confocal laser scanning microscopy (TCS SP2, Leica Microsystems, Germany) or Nikon C1Si (Nikon Imaging Facility, Heidelberg). Images were converted to Photoshop CS format (Adobe System, Inc.), pseudo-colored and merged.

### Protein extraction and immunoprecipitation assay

For cytoplasmic and nuclear protein extracts, embryos were de-chorionated, snap frozen in liquid N2, homogenized in buffer A (10 mM Hepes pH 7.9, 10 mM KCl, 1.5 mM MgCl_2_, 1 mM EDTA, 0.1 mM EGTA, 1 mM DTT, 0.34 M Sucrose, 10% w/v glycerol, 1 mM PMSF, 1× Roche complete proteinase inhibitors). Larvae (collected according to instar stage) and adult flies (dissected in PBS) were processed as described for embryonic tissue. After homogenization using a syringe (25 G), Triton-X-100 was added to 0.1% and proteins were extracted at 4°C for 30 minutes. The homogenate was filtered through gaze and processed as described previously [43]. For whole cell protein extracts, tissues were collected, snap frozen in liquid N_2_ and homogenized in IP-buffer (25 mM Tris, pH 8.0, 27.5 mM NaCl, 20 mM KCl, 25 mM sucrose, 10 mM EDTA, 10 mM EGTA, 1 mM DTT, 10% (v/v) glycerol, 0.5% Nonidet P40, 1 mM PMSF, 1× Roche complete proteinase inhibitors). To obtain soluble protein, extracts were spun at 16.000 g for 30 minutes at 4°C. For immunoprecipitation experiments, Protein A beads (GE Healthcare) were incubated with 3 µg anti-Dnmt2 antibodies and 1 µg/ml insulin (Sigma) for 1 hour at 4°C, washed 3×5 minutes in IP-buffer before whole cell protein extracts were added to the beads for 3 hours at 4°C. Peptide block was added in parallel at a concentration of 150 ng/ml to control for specificity of the IP-reaction. Beads were collected by centrifugation and washed 4 times 10 minutes at 4°C with IP-buffer, followed by 4 minutes at 94°C in SDS-sample buffer and Western analysis.

### Sub-cellular fractionation

Tissue was homogenized in hypotonic buffer A (10 mM Hepes pH 7.9, 10 mM KCl, 1.5 mM MgCl_2_, 0.34 M sucrose, 1 mM DTT, 1 mM PMSF, 1× Roche complete proteinase inhibitors). Lysed extracts were cleared of debris by low speed centrifugation at 400 g for 10 min at 4°C. Supernatants were spun at 1.700 g for 10 min 4°C to obtain crude nuclei. Crude nuclei were resuspended in buffer A and spun through a sucrose cushion containing 15 mM Tris pH 7.5, 60 mM KCl, 15 mM NaCl, 5 mM MgCl_2_, 1 M sucrose, 0.5 mM DTT, 1 mM PMSF, 1× Roche complete proteinase inhibitors) at 16.000 g for 30 minutes at 4°C. For whole nuclear protein extraction, nuclei were homogenized in urea extraction buffer (10 mM Tris pH 8, 100 mM NaH_2_PO4, 8 M urea) for 10 minutes at room temperature (RT), spun at 16.000 g for 10 minutes at RT and analyzed by Western blot. Nucleoli isolation was performed as described previously [44]. For nucleosomal extraction, purified nuclei were resupended in buffer A containing 1 mM CaCl_2_, diluted to a concentration of 200 µg/ml chromatin. MNase was added to 10 U/50 µg chromatin, incubated for 8 min at 37°C and stopped by addition of EDTA to 10 mM. Nuclei were pelleted and resuspended in 0.2 mM ice-cold EDTA, pH 7.0 for 1 hour (this hypotonic treatment forces nucleosomes into the supernatant). Remaining chromatin was pelleted by centrifugation and supernatant and nuclear pellet subjected to SDS-PAGE. For nuclear matrix extraction, purified nuclei were resupended in cytoskeletal buffer (CSK-buffer: 10 mM Pipes, pH 6.8, 100 mM NaCl, 300 mM sucrose, 3 mM MgCl_2_, 1 mM EGTA, 1× Roche complete proteinase inhibitors, 1 mM PMSF, 1 mM DTT, and 0.5% Triton X-100. Chromatin was solubilized by DNA digestion with 1 mg/ml of RNase-free DNAase I in CSK buffer plus proteinase inhibitors for 15 minutes at 37°C. Ammonium sulphate was added to 0.25 M and, after 5 minutes at 4°C, samples were centrifuged. The pellet was further extracted with 2 M NaCl in CSK buffer for 5 minutes at 4°C. The remaining pellet was solubilized in urea extraction buffer (see above) and was considered the nuclear matrix-containing fraction.

### Live imaging analysis

Staged collections were de-chorionated, thoroughly washed, aligned on a cover slip and overlaid with voltalef 10 S oil. For time-lapse analysis, images were acquired with a confocal laser scanning microscopy (TCS SP2, Leica Microsystems, Germany) using the 488 and 561 nm laser lines at low power (1–5%). Recording was carried out at 1 minute intervals and data was analyzed using ImageJ software.

## Supporting Information

Figure S1 Overexpression of EGFP does not lead to mitotic accumulation of EGFP. (A) Actin5C-GAL4 driven UAS-EGFP expression in the embryonic neuroectoderm shows no signs of a mitotic accumulation of EGFP at chromatin (prophase-upper panel, metaphase-lower panel). Scale bar: as in Fig. 4B
(0.76 MB TIF)

Movie S1 Live imaging of nuclear division cycles 10-11 of an embryo expressing pUbq-Dnmt2-EGFP and His2Av-mRFP1. Embryos were mounted on an open coverslip and viewed by dual wavelength time-lapse LSCM. Frames were recorded in 1 min intervals and images were merged with Dnmt2-EGFP in green and His2A-mRFP1 in red. Scale: as in Fig. 5A
(1.83 MB MPG)
