# Supplementary figures and images for: The Drosophila Cytosine-5 Methyltransferase Dnmt2 Is Associated with the Nuclear Matrix and Can Access DNA during Mitosis
Source: PLoS One. 2008 Jan 9;3(1):e1414. doi: 10.1371/journal.pone.0001414 (PMC2169302; doi:10.1371/journal.pone.0001414)

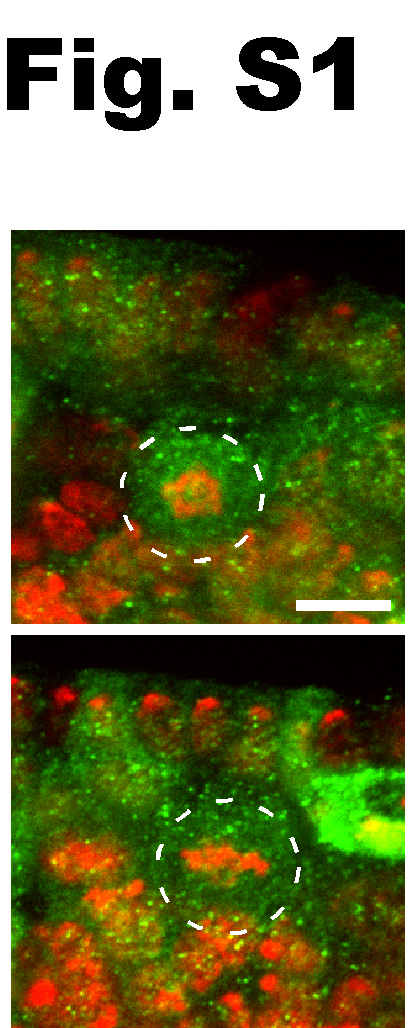

Supplement: Figure S1 — Overexpression of EGFP does not lead to mitotic accumulation of EGFP. (A) Actin5C-GAL4 driven UAS-EGFP expression in the embryonic neuroectoderm shows no signs of a mitotic accumulation of EGFP at chromatin (prophase-upper panel, metaphase-lower panel). Scale bar: as in Fig. 4B (0.76 MB TIF) [file pone.0001414.s001.tif]
